# Role of Histone Modifications in Kidney Fibrosis

**DOI:** 10.3390/medicina60060888

**Published:** 2024-05-28

**Authors:** Shengyu Pan, Tianhui Yuan, Yuqi Xia, Weimin Yu, Xiangjun Zhou, Fan Cheng

**Affiliations:** Department of Urology, Renmin Hospital of Wuhan University, Wuhan 430060, China; 2016283020168@whu.edu.cn (S.P.); whuyth@foxmail.com (T.Y.); xiayuqi1994@whu.edu.cn (Y.X.); ywm.com.cn@163.com (W.Y.)

**Keywords:** histone modifications, histone methylation, histone acetylation, renal fibrosis, epigenetic

## Abstract

Chronic kidney disease (CKD) is characterized by persistent kidney dysfunction, ultimately resulting in end-stage renal disease (ESRD). Renal fibrosis is a crucial pathological feature of CKD and ESRD. However, there is no effective treatment for this condition. Despite the complex molecular mechanisms involved in renal fibrosis, increasing evidence highlights the crucial role of histone modification in its regulation. The reversibility of histone modifications offers promising avenues for therapeutic strategies to block or reverse renal fibrosis. Therefore, a comprehensive understanding of the regulatory implications of histone modifications in fibrosis may provide novel insights into more effective and safer therapeutic approaches. This review highlights the regulatory mechanisms and recent advances in histone modifications in renal fibrosis, particularly histone methylation and histone acetylation. The aim is to explore the potential of histone modifications as targets for treating renal fibrosis.

## 1. Introduction

Chronic kidney disease (CKD) has become a global public health issue due to its high morbidity and mortality rates [1]. In 2017, CKD was responsible for a staggering 1–2 million deaths, with a prevalence of approximately 9.1 percent on a global scale [2]. A large proportion of CKD cases eventually proceed to end-stage renal disease (ESRD). Alarmingly, recent projection analyses suggest that CKD-related fatalities are poised to escalate dramatically, reaching a daunting 3.1 million individuals worldwide by the year 2040 [3]. Presently, the principal clinical treatments for severe CKD or ESRD encompass dialysis and kidney transplantation [4]. These treatment options not only profoundly impact patients’ quality of life but also impose an exorbitant economic burden on public healthcare systems [5,6]. Renal fibrosis is a pivotal pathological mechanism and a prognostic hallmark in CKD and ESRD [7]. The effective inhibition or reversal of fibrosis can markedly improve CKD patients’ survival and attenuate the progression from CKD to ESRD. Consequently, a complete understanding of the mechanisms of renal fibrosis holds the potential to explore novel therapeutic approaches.

Renal fibrosis encompasses two principal categories: glomerulosclerosis and renal interstitial fibrosis. Renal interstitial fibrosis represents an exuberant wound-healing response that occurs following diverse renal injuries, ultimately culminating in renal scarring. This progression leads to a gradual and irrevocable deterioration of renal architecture. Sustained damage causes an extracellular matrix (ECM) accumulation, leading to complete renal failure. Therefore, renal interstitial fibrosis emerges as the ultimate outcome in virtually all instances of CKD. Accumulating evidence underscores that the genesis of renal fibrosis is rooted in the fibrogenic niche, a distinct microenvironment that catalyzes myofibroblast activation [8,9,10].

**Figure 1:**
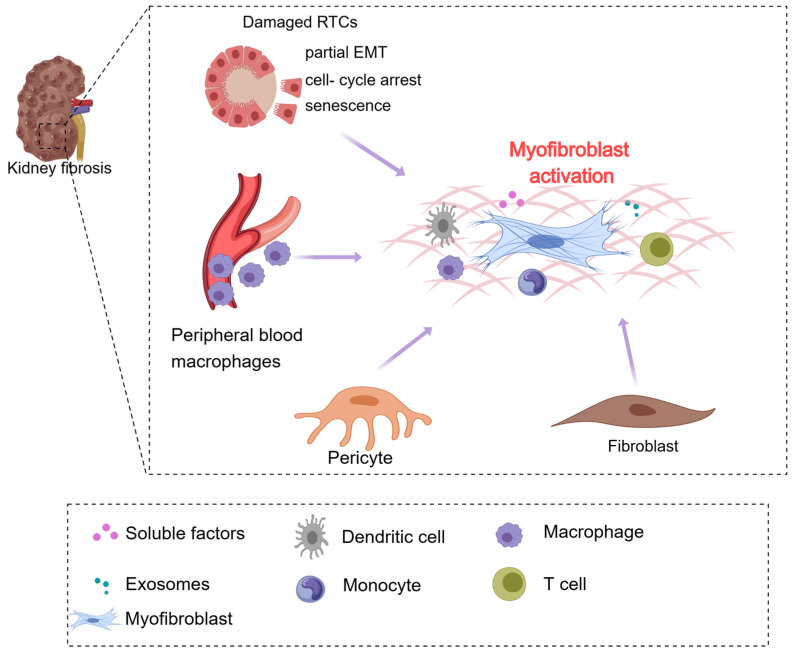
Origin and activation of myofibroblasts. The damaged renal tubular epithelial cells undergo partial EMT, cell cycle arrest, and senescence. They also secrete large amounts of pro-inflammatory, pro-fibrotic factors, chemokine, and growth factors to activate myofibroblasts and accumulate ECM. Additionally, renal intrinsic pericytes, fibroblasts, and peripheral blood-derived macrophages are major sources of myofibroblasts. Moreover, immune cells are significantly infiltrated in the renal interstitium. Abbreviations: partial epithelial–mesenchymal transition (partial EMT).

The fibrogenic niche encompasses a myriad of constituents, comprising renal tubular cells (RTCs), pericytes, vascular endothelial cells, stromal fibroblasts, inflammatory cells, extracellular vesicles, soluble factors, and the ECM network (Figure 1). The initiation and advancement of fibrosis involve intricate interplays among these diverse elements within the fibrogenic niche, culminating in a complex array of pathological alterations. These changes include partial epithelial–mesenchymal transition (partial EMT), pro-fibrotic factor and extracellular vesicle secretion, myofibroblast activation, cell cycle arrest, inflammatory cell infiltration, and metabolic reprogramming. Furthermore, macrophages can promote renal fibrosis through M2 polarization and macrophage-to-myofibroblast transformation (MMT) [11,12,13,14]. MMT emerges as a potentially pivotal predictor of the transition from chronic inflammation to pathological fibrosis [14].

CKD results from a combination of genetic and epigenetic regulators. Genetic variations determine an individual’s susceptibility to this disease [15,16]. These genetic variants are most commonly single nucleotide polymorphisms (SNPs). Although most SNPs have little effect on gene expression activity, some can exert biological effects through epigenetic regulation [17]. Epigenetic regulation entails the chemical modification of nucleic acids and histones, leading to alterations in chromatin structure while preserving the underlying nucleotide sequence. This complex process ultimately results in changed gene expression and stands as the primary regulatory mechanism governing cellular responses to environmental changes [18,19]. With the rapid development of epigenetics, it combines genetic variation with environmental exposure [20]. The more current view is that epigenetics may mediate this interaction to a significant extent [21]. This idea has become quite plausible, with many studies confirming that most genetic variation is mediated by the epigenome [17,22]. Liu et al. found that DNA methylation mediated a higher proportion of heritability associated with renal function than gene expression [17]. Additionally, DNA methylation-mediated heritability was distinctly tissue-specific, with significant enrichment observed in kidney-specific enhancer regions. Common epigenetic mechanisms encompass DNA methylation, histone modification, and non-coding RNA (ncRNA) regulation. A growing body of research underscores the pivotal contribution of epigenetic inheritance to the onset of fibrosis [23,24,25]. Meanwhile, numerous studies have demonstrated that various factors, including inflammation, injury, and altered metabolic status, can control genomic epigenetic reprogramming and further promote the progression of kidney disease. Among these epigenetic regulatory mechanisms, histone modification has garnered significant attention in recent studies. Notably, modifications of histones exhibit a reversible nature, hinting at their potential as therapeutic targets for renal fibrosis. Consequently, achieving a comprehensive understanding of the mechanisms and functions of histone modifications in the development of fibrosis assumes paramount significance in the quest for safer and more efficacious therapies against renal fibrosis. This review primarily focuses on elucidating the role of histone modifications in renal fibrosis while also delving into recent advances in this field. Furthermore, it contemplates the feasibility of targeting histone modifications as a novel therapeutic strategy for addressing these conditions.

## 2. Histone Modification

The nucleosome, serving as the fundamental unit of chromatin, is composed of histones and DNA. At its core lies a histone octamer, comprised of two H2A, H2B, H3, and H4 histone proteins, enveloped by approximately 147 base pairs of DNA [26]. Histone post-translational modifications (HPTMs) can occur in both the N- and C-terminal tails. However, it is noteworthy that the N-terminal portion is particularly susceptible to modifications, owing to its non-participation in nucleosome assembly and its extension outward from the nucleosome structure [27]. Histones H3 and H4 possess tails longer than those of H2A and H2B, making them more susceptible to HPTMs.

**Figure 2:**
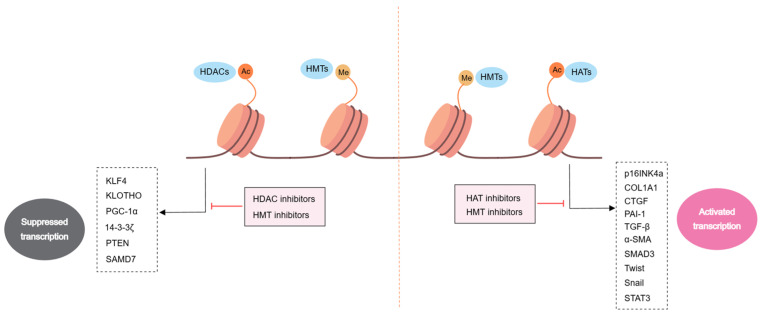
Histone methylation and histone acetylation in renal fibrosis. HMTs (e.g., H3K4me1, H3K4me3) and HATs activate the expression of pro-fibrotic-related proteins. In contrast, HMTs (e.g., H3K27me3, H3K79me2) and HADCs inhibit the expression of antifibrotic-associated proteins. Abbreviations: Alpha-Smooth-Muscle Actin (α-SMA); Collagen-1α (COL1A1); Cellular Communication Network Factor 2 (CTGF); Kruppel-Like Factor 4 (KLF4); Peroxisome Proliferator-Activated Receptor Gamma Coactivator 1 Alpha (PGC-1α); Phosphatase And Tensin Homolog (PTEN); Plasminogen Activator Inhibitor 1 (PAI-1); Transforming Growth Factor Beta (TGF-β); Signal Transducer and Activator of Transcription 3 (STAT3); histone methyltransferase (HMT); histone acetyltransferase (HAT); histone deacetylase (HDAC).

Presently, an extensive array of more than 25 distinct forms of histone modifications has been identified. Nonetheless, the histone modifications that have received extensive research attention encompass primarily methylation, acetylation, phosphorylation, and ubiquitination. Of these, methylation and acetylation have emerged as the most comprehensively investigated. Histone modifications are reversible covalent changes governed primarily by histone-modifying enzymes and their corresponding cofactors. These include ‘writers’, enzymes responsible for catalyzing the establishment of specific types of HPTMs; ‘erasers’, enzymes tasked with removing HPTMs; and ‘readers’, enzymes designed to recognize HPTMs. HPTMs regulate gene transcription via their influence on chromatin conformation. Notably, the deregulation of HPTMs is inextricably linked to the development of renal fibrosis, highlighting the pivotal role of HPTMs in this context (Figure 2).

## 3. Histone Methylation

**Figure 3:**
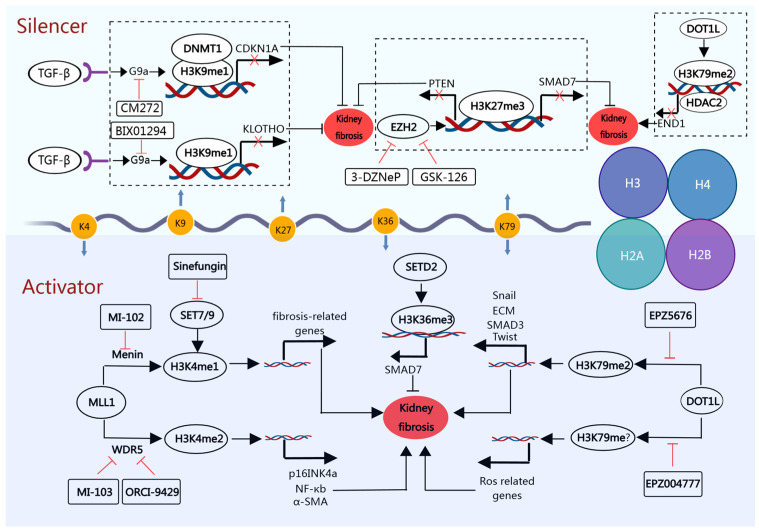
Histone methyltransferases (HMTs) are involved in regulating renal fibrosis. Abbreviations: Mixed-lineage leukemia 1 (MLL1); WD repeat domain 5 (WDR5); SET Domain-Containing Protein 7 (SET7/9); Alpha-Smooth-Muscle Actin (α-SMA); extracellular matrix (ECM); nuclear factor kappa B (NF-κB); Euchromatic Histone Lysine Methyltransferase 2 (G9a); Enhancer of zester homolog 2 (EZH2); Phosphatase And Tensin Homolog (PTEN); DOT1-Like Histone Lysine Methyltransferase (DOT1L); SET Domain-Containing 2 (SETD2); Transforming Growth Factor Beta (TGF-β); reactive oxygen species (ROS); Endothelin 1 (EDN1).

Histone methylation is a crucial post-translational modification (PTM) that regulates many biological processes. While it was initially thought to be permanent, the discovery of histone demethylases transformed our understanding of this process. This led to the realization that histone methylation is reversible [28,29]. Lysine and arginine residues within the tails of histones H3 and H4 are common sites for methylation modifications. Prominent among these are lysine methylation sites such as H3K4, H3K9, H3K27, H3K36, and H3K79, each exhibiting three distinct variations: monomethylation, dimethylation, and trimethylation. Arginine methylation, on the other hand, manifests in three forms: monomethylation, symmetric dimethylation, and asymmetric dimethylation. It is worth noting that, unlike other HPTMs, the abundance and specific location of histone methylation marks can profoundly influence the transcriptional fate, either activating or repressing gene expression. Histone methylation is dynamically regulated by both histone methyltransferases (HMTs) (WRITERS) and histone demethylases (ERASERS) and exerts its effects through the binding of readers containing specific structural domains [30]. HMTs are the primary modality regulating histone methylation and can be classified into lysine methyltransferases (KMTs) and arginine methyltransferases (PRMTs). Histone demethylases (HDMTs) can be categorized into lysine-specific demethylases (LSDs) and JMJC structural domain-containing family (JMJD). An emerging body of research highlights the central role of HMTs (Figure 3) and demethylases in the intricate regulation of renal fibrosis, underscoring their importance.

## 4. KMTs and PRMTs in Renal Fibrosis

MLL1, a member of the SET1 family, catalyzes the mono-, di-, and trimethylation of H3K4. The SET1 family has weak enzymatic activity, but interactions with WDR5, RbBP5, Ash2L, and Dpy30 (WRAD) subunits significantly enhanced the methyltransferase activity of its SET structural domain [31,32,33,34]. Zou et al. showed that the expression of MLL1-menin and its specific substrate H3K4me1 was increased in a unilateral ureteral obstruction(UUO) model and that MI-503 (an MLL1-menin inhibitor) downregulated the expression of H3K4me1 and inhibited RTC EMT and myofibroblast activation and attenuated renal fibrosis [35]. MM-102 and OICR-9429 inhibited MLL1/WDR5 binding and suppressed p16INK4a expression by downregulating H3K4me3, attenuating renal fibrosis and senescence after renal ischemia-reperfusion injury (IRI) [36].

SET7/9 facilitates the methylation of histone H3 at lysine 4 (H3K4), giving rise to the formation of H3K4 monomethylation (H3K4me1). Recent evidence highlights the critical role of SET7/9 in renal fibrosis. It has been reported that increased SET7/9 expression in rat mesangial cells under TGF-β stimulation promotes the expression of P21 [37] and ECM-related genes (COL1A1, CTGF, PAI-1) [38] by recruiting H3K4me1 enriched in the promoter regions of these genes, thereby mediating renal fibrosis. SASAKI et al. showed that SET7/9 is regulated by the TGF-β/SMAD3 pathway in the UUO model and that either si-SET7/9 or the small molecule inhibitor sinefungin downregulates the expression of α-SMA and ECM proteins by inhibiting H3K4me1 [39].

G9a is a crucial enzyme for H3K9 mono- and demethylation [40] and also catalyzes trimethylation of H3K9 [41]. In a mouse model of UUO, stimulation of TGF-β leads to upregulation of G9a expression via SMAD9 activation. Subsequently, G9a suppresses the level of KLOTHO via H3K9me1 and induces kidney fibrosis. However, BIX01294 (a G9a inhibitor) attenuated renal fibrosis by restoring KLOTHO expression via inhibiting H3K9me1 in vivo and in vitro [42]. In addition, studies in human kidney biopsy specimens have shown a negative correlation between G9a expression and the extent of kidney fibrosis, as well as KLOTHO protein expression [42]. Recent research has demonstrated that CM272, a dual inhibitor of G9A and DNMT1, has shown remarkable promise in the reduction in liver fibrosis. Its mechanism of action involves inhibition of TGF-β-induced metabolic reprogramming and restoration of gluconeogenesis and mitochondrial function. CM272 achieves these effects by reducing H3K9me2 enrichment within the FBP1 and PGC-1α promoter region and alleviating DNA methylation. Meanwhile, CM272 was found to inhibit the HIF pathway, which is expected to be an effective target for the treatment of renal fibrosis [43]. Yang et al. further demonstrated that CM272 effectively attenuates renal fibrosis by reducing H3K9me2 accumulation and DNA methylation in the promoter region of CDKN1A, thereby attenuating cell cycle arrest [44]. These results suggest that G9a plays an important role in renal fibrosis.

EZH2, the most widely studied histone methyltransferase, plays an important role in normal kidney development and disease. EZH2 inhibition reverses the fibrotic phenotype. Zhou et al. found that EZH2 inhibition of SMAD7 expression via H3K27me3 activated the TGF-β-SMAD3 pathway to promote renal fibrosis in UUO. Furthermore, EZH2 promoted renal fibrosis by inhibiting PTEN expression and activating STAT3 and ERK1/2 phosphorylation [45]. Additionally, EZH2, through activation of the AKT/mTOR and β-catenin pathways, promotes RTCs EMT and cell cycle arrest to propel renal fibrosis [46]. In renal IRI and folate-induced injury models, EZH2-mediated H3K9me3 enrichment in the PTEN promoter region effectively restricts PTEN transcription. Kidney fibrosis caused by PTEN inhibition is rescued by inhibiting EZH2. This study also found that in RAW cells, inhibition of EZH2 suppressed the p-Stat6 and PI3K/AKT pathway, inhibited M2 macrophage polarization, and reduced MMP-9 secretion, providing a countermeasure against renal fibrosis [47]. Compounds such as gambogenic acid [48], salvianolic acid B [49], and emodin [50] have emerged as potential therapeutic agents against renal fibrosis due to their ability to inhibit EZH2 activity and its associated epigenetic marker, H3K9me3. In summary, EZH2 appears to play a multifaceted role in promoting renal fibrosis through several complex pathways. Targeted inhibition of EZH2 is emerging as a promising therapeutic avenue to attenuate renal fibrosis with potential clinical benefits.

DOT1L stands as the sole known histone H3K79 methyltransferase capable of catalyzing H3K79 in mono-, di-, and trimethylation forms [51,52]. Notably, H3K79 methylation is prominently enriched within the coding regions of genes closely associated with transcriptional activation [53]. Beyond its role in transcriptional regulation, DOT1L has a multifaceted impact on various cellular processes, encompassing DNA repair, cell differentiation, and cell cycle regulation [54,55]. A study by Liu et al. has unveiled intriguing insights into the role of DOT1L in renal fibrosis. Inhibition of DOT1L was found to effectively curtail myofibroblast cell activation and RTCs’ EMT, concomitantly upregulating renal fibroprotective factors such as PTEN, KLOTHO, and SMAD7 [56]. Within an IRI model, EPZ004777, a DOT1L inhibitor, demonstrated the capacity to suppress PI3K/AKT-mediated reactive oxygen species (ROS) production, ultimately mitigating renal fibrosis [33]. However, the role of Dot1L in renal fibrosis remains controversial. Interestingly, studies in the UUO and diabetic nephropathy (DN) mouse models have positioned DOT1L as an antifibrotic molecule, primarily by inhibiting Edn1 expression. DOT1L knockdown removes H3K79me2 from the Edn1 promoter region and recruits HDAC2, increasing H3 acetylation and Edn1 expression [57]. These divergent findings highlight the complex and multifaceted nature of DOT1L’s functions and suggest that its modulation of different targets and pathways may lead to different outcomes in renal fibrosis.

PRMT1, a member of the type I arginine methyltransferases, exhibits the capacity to asymmetrically methylate histone substrates, with H4R3 being its predominant substrate [58,59,60]. The asymmetric methylation of H4R3 serves as a pivotal epigenetic hallmark associated with transcriptional activation [61]. PRMT1 induces endoplasmic reticulum (ER) stress and EMT to promote renal fibrosis in the DN model [62]. In the UUO model, PRMT1 contributes to renal fibrosis by modulating the TGF-β/SMAD3 pathway, a process attenuated by the inhibitory effects of AMI-1 [63]. Interestingly, and in apparent contradiction, Wu et al. reported an ameliorating effect of PRMT1 and the marker AMDA on renal fibrosis. This improvement is attributed to inhibiting nitric oxide (NO) levels in HK-2 cells, whereas the inhibitor PT1001B, which targets PRMT1, exacerbates renal fibrosis [64]. This dual role of PRMT1 underlines the complex nature of its involvement in renal fibrosis and highlights the need for careful consideration in the selection of PRMT1 inhibitors, requiring a comprehensive understanding before definitive conclusions can be drawn regarding the therapeutic potential of PRMT1 inhibitors in the context of renal fibrosis.

## 5. Histone Demethylases in Kidney Fibrosis

LSD1 specifically demethylates H3K4me1/2 and H3K9me1/2 and represses or promotes gene transcription [65]. LSD1 is highly expressed in UUO and DN and promotes involvement in renal fibrosis through multiple pathways, including EMT [66,67], cell cycle arrest [66], myofibroblast activation [66,68], and inflammation [69]. Zhang et al. first found that the LSD1-14-3-3ζ-PKCα axis regulates the activation of STAT3 and AKT pathways, which mediates renal fibrosis, and ORY1001 reverses this process [66]. LSD1 inhibitors are being tested in clinical trials for solid tumors and have shown promising efficacy. However, there is a lack of clinical trials investigating the use of LSD1 inhibitors in fibrosis. Therefore, further high-quality basic and clinical studies are needed to validate the role of LSD1 inhibitors in renal fibrosis [70].

JMJD3, a member of the UTX/UTY JmjC domain protein subfamily, has the capacity for H3K27me2/3 demethylation. Recent studies have shown that bone marrow-derived fibroblasts [71,72] and M2 MMT play an important regulatory role in fibrosis [13,14,73,74]. Central to these regulatory processes is the JMJD3/IRF4 axis, a key orchestrator governing the aggregation of bone marrow-derived fibroblasts and the activation of MMT. Pharmacological inhibition of JMJD3 with GSK-J4 attenuates renal fibrosis by inhibiting M2MMT [75,76]. Ying et al. found that the knockdown of JMJD3 in myeloid cells could inhibit hypertension-induced renal inflammation and fibrosis [77]. Nevertheless, in UUO and subtotal nephrectomy (SNx) models, Chao et al. found that JMJD3 had inhibitory effects on the TGF-β/SMAD3 and Notch pathways associated with fibrosis. Furthermore, JMJD3 activation led to the stimulation of PTEN, effectively inhibiting the phosphorylation of AKT and ERK1/2, thereby impeding the progression of fibrosis. GSK-J4 paradoxically exacerbated renal fibrosis in these specific models [78]. Considering that JMJD3 plays a dual role in renal fibrosis, JMJD3-targeted therapies should be administered according to the type of disease in the clinical setting, and more in-depth studies are also needed to explore the mechanisms behind the differences. The mechanisms of histone demethylases in renal fibrosis are summarized in Table 1.

## 6. Histone Acetylation

Acylation is transferring and adding an acetyl moiety of acyl-CoA to the protein N-terminus or lysine residue. Acylation is a highly dynamic and reversible PTM, which plays a significant role in biological processes such as inflammation, immunity, and metabolism. Acyl-CoA, a critical metabolite, is an essential substrate for acetylation. Consequently, the cell’s metabolic state significantly regulates the targets and kinetics of acetylation [79]. Histones are among the most highly acetylated proteins and are dynamically regulated by histone acetyltransferases (HATs) and histone deacetylases (HDACs) [80]. HATs mediate histone acetylation by catalyzing the transfer of an acetyl group to a lysine residue. In contrast, HDACs remove the acetyl group from histone lysine residues. Histone acetylation is primarily associated with the activation of gene transcription [81]. Acetylation neutralizes the intrinsic positive charge of lysine residues, reducing the positive charge of histone proteins and their affinity for negatively charged DNA molecules, leading to the chromatin structure’s relaxation, which allows transcriptional regulators to bind and promote gene expression. In addition, as suggested by the histone code hypothesis, histone modifications are local information carriers that drive downstream transcriptional responses by recruiting ‘reader’ proteins for binding [82]. Thus, histone acetylation may also fulfill its function of regulating gene transcription by recruiting ‘reader’ proteins. Several protein structural domains are known to have the ability to recognize or ‘read’ acetylated lysine residues precisely. These include the bromodomains, YEATS domains, and double plant homeodomain (PHD) finger domains, with the bromodomain being the most extensively studied [83,84,85,86]. Genome-wide histone acetylation has been shown to enhance correlations between transcriptional regulatory regions or between these regions and transcriptional activators. One of the hallmarks of this increased enhancer activity is increased levels of H3K27 acetylation, which promotes transcription by recruiting bromodomain-containing proteins, including BRD4 and the transcription initiation factor TFIID subunit 1 (TAF1) [87]. Beyond its role in gene expression, acetylation plays a pivotal role in fundamental cellular processes including metabolism, RNA processing, translation, protein folding, chromatin organization, protein degradation, and cytoskeletal dynamics [88]. Many recent studies have identified an important role for histone acetylation in renal fibrosis, and we will next focus on the role of HATs and HDACs in renal fibrosis.

## 7. HATs in Kidney Fibrosis

HATs, a diverse group of enzymes, are broadly categorized into three groups: P300/CBP, the MYST family, and the GNAT superfamily. MYST proteins include Esa1, Sas2, Tip60, MOF, MOZ, and MOR. Members of the GNAT superfamily include Gcn5, PCAF, Elp3, Hpa2, and Hat1.

P300/CBP is the most widely studied HAT. P300/CBP proteins not only have intrinsic HAT activity but also promote gene transcription by binding to other transcription factors (e.g., STAT proteins, AP1, and NF-κB) [89]. In the regulation of fibrosis, CBP/P300 emerges as a pivotal regulator [90], fostering renal fibrosis progression through the facilitation of ECM synthesis, generation of ROS, and promotion of inflammation [91,92]. A wealth of studies collectively underscores the potential therapeutic impact of targeting P300 in renal fibrosis. L002, a FATP300 inhibitor, attenuates renal fibrosis by inhibiting H4 acetylation, thereby reducing the expression of COL1A1 and COL4A3 [91]. Gong et al. found that knocking down EP300 effectively inhibited HIF-2α expression, thereby attenuating renal fibrosis in vivo and in vitro in DN [93]. C646 also showed a remarkable ability to attenuate renal fibrosis through multiple mechanisms in the DN model. Its effects are characterized by inhibition of H3K27 acetylation, concomitant suppression of NF-κB and STAT3 transcriptional activity, and attenuation of the expression of inflammatory mediators (including MCP-1, TNFα, NOS2, ICAM-1, VCAM-1, and E-selectin), along with concomitant dampening of pro-fibrotic molecules [92]. Moreover, C646 demonstrated efficacy in attenuating peritoneal fibrosis induced by EMT in peritoneal cells by inhibiting the TGF-β1/SMAD3 pathway [94].

PCAF, a constituent of the GNAT superfamily, exhibits elevated expression in the UUO model, fostering renal fibrosis through the mediation of inflammation, ROS, and EMT. Furthermore, PCAF facilitates an escalation in nuclear NF-κB expression and concurrently induces a reduction in Nrf2 expression. The compound Garcinol inhibits the levels of PCAF and H3K9ac, resulting in decreased nuclear NF-κB expression and increased nuclear Nrf2 expression. As a result, renal fibrosis is reversed [95].

TIP60, a member of the MYST family, remains unexplored in the context of renal fibrosis. Nevertheless, a substantial body of research underscores the pivotal role of TIP60 in diverse biological processes such as metabolism, aging, inflammation, and autophagy [96,97,98,99]. Studies have shown that the knockdown of TIP60 promotes ECM remodeling and attenuates cardiac fibrosis [96]. Thus, TIP60 may be a potential target for the treatment of renal fibrosis and warrants further investigation. The functions of HATs in renal fibrosis are summarized in Table 2.

## 8. HDACs in Kidney Fibrosis

The 18 histone deacetylases (HADCs) under scrutiny can be systematically categorized into four distinct classes, discernible by their structural characteristics and subcellular localization [100,101,102]. Class I comprises Rpd3-like proteins, which encompass HADC1,2,3, and HDAC8. Moving to class II, it is comprised of Hda1-like proteins embracing HADC4,5,6,7, HADC9, and HADC10, with class IIa featuring HDAC4, 5, 7, and 9, and class IIb showcasing HDAC6 and 10. Class III is comprised of Sir2-like proteins, namely sirt1–7, while class IV stands as a solitary entity housing only one member, HDAC11. Classes I, II, and IV are Zn^2+^-dependent histone deacetylases (HDACs) and class III contains NAD+-dependent Sirtuins.

In vitro experiments have unveiled the pivotal involvement of class 1 HDACs in the regulation of ECM and EMT. Notably, the application of MS275, an inhibitor targeting HDA1,2,3 (excluding HDAC8), exhibited a marked reduction in the expression of COL1 and the reversion of E-cadherin proteins. PCI34051 (a specific inhibitor of HDAC8) suppressed ECM expression but not EMT [103]. In the UUO model, HDAC1 and HDAC2 expression was upregulated, and TSA, valproate, and knockdown of HDAC1 or HDAC2 attenuated CSF-1 expression and macrophage infiltration, thereby inhibiting fibrosis [104]. In an aldosterone-induced CKD model, elevated HDAC1 expression inhibits H3K9 acetylation, leading to the downregulation of KLOTHO protein expression, which promotes renal fibrosis [105]. HDAC2 is highly expressed in diabetic kidney disease (DKD) and mediates the development of renal fibrosis through a variety of mechanisms [106,107,108]. Concurrently, HDAC3 is upregulated by the TGF-β/SMAD pathway in mouse models of UUO, aristolochic acid nephropathy (AAN) [109], and adenine-induced CKD [110]. In the UUO model, the HDAC3 inhibitor RGFP966 inhibits renal fibrosis by restoring KLOTHO expression through the inhibition of KLOTHO transcriptional regulators (Ncor and NF-κb) [109]. However, in the adenine CKD mouse model, the application of trichostatin A and the specific knockdown of HDAC3 facilitate PPARγ acetylation, leading to the upregulation of KLOTHO protein and the concurrent inhibition of renal fibrosis [81]. Hu et al. demonstrated that in the hyperuricemic renal fibrosis model, the knockdown of HDAC3 attenuated renal fibrosis via miR-19b-3p/SF3B3 [111]. HDAC8, identified as upregulated in the UUO model, is a promoter of renal fibrosis by influencing EMT-related pathways, inducing cell cycle arrest and suppressing the expression of BMP-7 and KLOTHO proteins. Importantly, the pro-fibrotic effects of HDAC8 are ameliorated by the application of PCI34051 [112]. These insights illuminate the intricate roles of class I HDACs in the molecular pathways underpinning renal fibrosis, thereby providing potential targets for therapeutic interventions in CKD.

As mentioned above, class II HDACs can be divided into classes IIa and IIb. Xiong et al. observed that the expressions of HDAC4, 5, 7, and 9 were elevated in the UUO model and predominantly localized in the cytoplasm of renal tubules, with HDAC4 exhibiting the most conspicuous increase [113]. Class IIa HDAC inhibitor MC1568 inhibits EMT-mediated renal fibrosis by inhibiting the activation of TGF-β/SMAD3 and the NF-κb signaling pathway, and upregulating the expression of BMP-7, KLOTHO protein [113]. Shen et al. found that knockdown of HDAC4 effectively inhibited renal fibrosis through the following mechanisms: the inhibition of EMT-related pathways (TGF-β/SMAD3, STAT3, ERK1/2 pathways), inhibition of cell cycle arrest, attenuation of apoptosis, and retention of KLOTHO protein [114]. Piceatannol may attenuate renal fibrosis by inhibiting HDAC4 and HDAC5 [115]. However, the specific roles and mechanisms of HDAC5, 7, and 9 in animal models of renal fibrosis remain elusive due to the absence of dedicated class IIa inhibitors. This limitation underscores the ongoing challenge in fully elucidating the nuanced contributions of these HDACs in the intricate landscape of renal fibrosis.

HDAC6, a class IIb HDAC expressed in the cytoplasm, is highly expressed in a variety of renal diseases and plays an important role in regulating inflammation, pro-fibrotic signaling pathways, and oxidative stress [116]. ACY-1215 (a selective HDAC6 inhibitor) could reduce renal fibrosis by inhibiting pro-fibrotic pathways (TGF-β/SMAD3, EGFR), myofibroblast activation, and inflammation [117]. CAY10603 (a specific inhibitor of HDAC6) attenuates renal fibrosis in DKD by inhibiting NLRP3 inflammasome activity in RTCs and macrophages [118].

In mammals, class III HDACs are NAD+-dependent deacetylases, exemplified by the sirtuins 1–7, with Sirt1 being particularly well-studied. Sirt1 plays a pivotal role in governing cellular metabolism, growth, aging, inflammation, and oxidative stress. Its significance in renal diseases is underscored by its demonstrated protective functions. In the UUO model, He et al. found that sirt1 knockdown exacerbated renal apoptosis, fibrosis, and ROS production. Conversely, SRT1720 (sirt1 agonist) attenuated renal apoptosis, fibrosis, and ROS [119]. Sirt1 exerts its antifibrotic effects through the deacetylation of various non-histone substrates, regulating senescence, metabolism, EMT, and autophagy. Targets of Sirt1 include HIF-1α [120], HIF-2α [121], SMAD3 [122], FOXO3 [123], P53 [124], and PGC-1α [125]. While the protective role of Sirt3 against fibrosis was initially identified in the heart, emerging evidence supports its protective role in renal fibrosis. Mechanistically, Sirt3 inhibits glycolysis [126,127], promotes oxidative phosphorylation [128], and deacetylates β-catenin [129], thereby restraining the expression of pro-fibrotic factors. Recent studies have shown that Sirt6 knockdown exacerbates renal fibrosis after UUO and that the protective mechanism of Sirt6 is the inhibition of β-catenin expression [130,131]. Mdl-800 (Sirt6 agonist) attenuates renal fibrosis and inflammation in UUO [130]. This expanding understanding of the roles of Sirt1, Sirt3, and Sirt6 in renal fibrosis provides potential avenues for targeted therapeutic interventions in kidney diseases.

HDAC11, the sole representative of the class IV HDACs, is distributed within the nucleus and cytoplasm of mitochondrial cells, exhibiting heightened expression in key tissues such as the heart, brain, kidney, smooth muscle, and skeletal muscle. HDAC11 has been identified as upregulated in the UUO model, where it exerts a pro-fibrotic influence by inhibiting KLF15. The application of Quisinostat, an HDAC11 inhibitor, has been demonstrated to effectively reverse renal fibrosis in this model [132]. Impaired fatty acid metabolism in renal tubular epithelial cells plays a pivotal role in renal fibrosis. Sun et al. demonstrated that HDAC11 knockdown activates the adiponectin-AdipoR-AMPK pathway and contributes to the reversal of hepatosteatosis [133]. Subsequent studies by Bi et al. revealed that HDAC11 promotes glycolysis through activation of the LBK1-AMPK pathway, thereby facilitating hepatocellular carcinoma recurrence, metastasis, and drug resistance [134]. These studies collectively highlight the significant regulatory function of HDAC11 in metabolic reprogramming. Therefore, further research is needed to confirm whether HDAC11 is involved in regulating metabolic reprogramming in CKD. These insights into the multifaceted functions of HDAC11 shed light on its potential as a therapeutic target in the intricate landscape of renal fibrosis and metabolic dysregulation. These novel insights into the functions of HDAC11 shed light on its potential as a therapeutic target for metabolic dysregulation in renal fibrosis. The functions of HADCs in renal fibrosis are summarized in Table 3.

In recent years, investigations have spotlighted acetylation as a pivotal epigenetic mechanism in renal fibrosis. Recognizing the significance of this process, the targeting of HATs or HDACs has emerged as a promising avenue for potential fibrosis treatment.

## 9. Conclusions and Future Perspectives

Fibrosis is the characteristic pathological alteration that occurs in CKD due to various etiologies. Although the pathogenesis of renal fibrosis remains largely unknown, knowledge of its initiation and progression have been greatly enhanced over the decades. Firstly, renal injury triggers inflammation with immune cell infiltration; secondly, RTC injury and endothelial cell shedding accelerate the release of pro-fibrotic mediators like growth factors, chemokines, and cytokines; thirdly, myofibroblast activation and excessive accumulation of ECM in the tubular mesenchyme occur due to an imbalance between ECM synthesis and degradation; fourthly, phenotypic alterations and irreversible loss of parenchymal cells take place; and fifthly, the reduction in kidney microvasculature leads to local hypoxia, hypoperfusion, and changes in metabolites.

Over the past decade, histone modifications have attracted widespread interest as diagnostic and prognostic indicators for renal fibrosis, as well as potential therapeutic targets. Renal fibrosis is influenced by histone modification at all stages. Therefore, the reversibility of epigenetics offers a positive aspect for treating the condition. Although few drugs have been approved for the clinical treatment of fibrosis, epigenetic drugs have been used in the clinical treatment of solid tumors. These epigenetic drugs include EZH2 inhibitors (tazemetostat), dual EZH1/EZH2 inhibitors (valemetostat), and HDAC inhibitors (vorinostat, belinostat, romidepsin, tucidinostat, and panobinostat) [135,136,137,138]. Notably, the therapeutic indications for HDAC inhibitors are expanding beyond oncology to non-oncology diseases, with clinical trials being progressively refined. Examples include trials for Alzheimer’s disease (NCT03056495), epilepsy (NCT03894826), and Crohn’s disease (NCT03167437). Clinical trials for renal fibrosis may, therefore, be anticipated in the near future.

This review highlights the significance of histone modifications in developing renal fibrosis. By summarizing data from numerous preclinical studies, it is demonstrated that drugs targeting dysregulated histone modification-associated enzymes (HMTs, HDMTs, HATs, HDACs) exhibit beneficial antifibrotic effects, offering new perspectives for treating renal fibrosis. Despite the promise of targeted histone modification as an effective target for the clinical treatment of renal fibrosis, its toxicities in the treatment of oncology patients remain a non-negligible problem. This may be related to the fact that the effects of targeted histone modifications are genome-wide and that most HDAC inhibitors have poor specificity. Therefore, developing highly selective targeted drugs and precise delivery systems (extracellular vesicles, nanoparticles, nanogels, etc.) will be the future direction of targeted histone modification to treat renal fibrosis.

## Figures and Tables

**Table 1:** Histone demethylases in kidney fibrosis.

Histone Demethylases	Interventions	HISTONE SITE	Model	Effects and Mechanisms	Ref.
LSD1	ORY-1001	H3K4me1, H3K4me2	UUO	Protective; EMT↓, renal fibroblast activation↓, TGF-β↓, SMAD3↓, p-STAT3↓, p-AKT↓, 14-3-3ζ↑	[66]
	LSD1 KO	Not mentioned	DN	Protective; Sirt3↑, TGF-β↓, SMAD3↓	[68]
JMJD3	JMJD3 KO, GSK-J4	Not mentioned	UUO, FA	Protective; M2 MMT↓, myeloid fibroblast activation↓, IRF4↓	[75]
	GSK-J4	H3K27me3	UUO	Protective; myeloid fibroblast activation↓, M2 macrophage polarization↓,	[76]
	JMJD3 KO, GSK-J4	H3K27me3	UUO, 5/6 SNx	Pro-fibrotic; TGF-β↑, SMAD3↑, Notch1↑, Notch3↑, SMAD3↓, p-AKT↑, p-ERK↑, SMAD7↓, PTEN↓	[78]

Abbreviations: Lysine Demethylase 1A (LSD1); epithelial–mesenchymal transition (EMT); Transforming Growth Factor Beta (TGF-β); Phosphorylated transducer and activator of transcription-3 (p-STAT3); Phospho-AKT Serine/Threonine Kinase 1 (p-AKT); Sirtuin 3 (Sirt3); Lysine Demethylase 6B (JMJD3); Macrophage-to-myofibroblast transformation (MMT); Interferon Regulatory Factor 4 (IRF4); Phosphatase And Tensin Homolog (PTEN); Notch Receptor 1 (Notch1); Notch Receptor 3 (Notch3); Phospho-Extracellular-regulated protein kinases (p-ERK); Unilateral ureteral obstruction (UUO); diabetic nephropathy (DN); Folic acid (FA); 5/6 nephrectomy (5/6 SNx).

**Table 2:** HAT inhibitors in renal fibrosis.

Interventions	Target	Model	Effects and Mechanisms	Ref.
L002	FATP300	Hypertensive cardio-renal fibrosis	Protective; H4ac↓,COL1A1↓, COL4A3↓,α-SMA↓	[91]
EP300 KO	EP300	DN	Protective; HIF-2α↓	[93]
C646	P300	DN	Protective; H3K27ac↓, ROS↓, fibronectin↓, NF-κb↓, STAT3↓	[92]
Garcinol	PCAF	UUO	Protective; H3K9ac↓,NF-κb↓,Nrf2↑,ROS↓	[95]

**Abbreviations:** E1A Binding Protein P300 **(P300,EP300);** Collagen-1α (COL1A1); Collagen Type IV Alpha 3 Chain **(COL4A3);** Alpha-Smooth-Muscle Actin (α-SMA); Hypoxia-inducible factor-2α (HIF-2α); reactive oxygen species (ROS); nuclear factor kappa B (NF-κB); Signal Transducer and Activator of Transcription 3 (STAT3); NFE2-Like BZIP Transcription Factor 2 (Nrf2); histone acetyltransferase (HAT); Unilateral ureteral obstruction (UUO); diabetic nephropathy (DN).

**Table 3:** HDACs in renal fibrosis.

Interventions	Target	Model	Effects and Mechanisms	Ref.
TSA	HDAC1,2	UUO	Protective; H3ac↓, CSF-1↓, macrophage infiltration↓	[104]
Sulforaphane	HDAC2	DN	Protective; H3K9ac↓, H3K14ac↓, BMP-7↑, ECM↓	[108]
RGFP966	HDAC3	UUO, AAN	Protective; H3K4ac↑, H3K9ac↑, H4K5ac↑, NF-κb↓, Ncor↓, KLOTHO↑	[109]
TSA	HDAC3	adenine CKD	Protective; KLOTHO↑, PPARγ acetylation↑	[110]
PCI34051	HDAC8	UUO	Protective; KLOTHO↑, BMP-7↑, EMT↓, cell cycle arrest↓	[112]
MC1568	IIa HDACs	UUO	Protective; EMT↓, BMP-7↑, KLOTHO↑,	[113]
Tasquinimod, HDAC4 KO	HDAC4	UUO	Protective; EMT↓, cell cycle arrest↓, KLOTHO↑, tubular cell apoptosis↓	[114]
Piceatannol	HDAC4,5	UUO	Protective; p-P38-MAPK↓	[115]
ACY-1215	HDAC6	UUO	Protective; H3ac↑, SMAD7↑, TGF-β↓, SMAD3↓, EGFR↓, p-STAT3↓, NF-κb↓	[117]
CAY10603	HDAC6	DKD	Protective; NLRP3↓	[118]
Quisinostat	HDAC11	UUO	Protective; KLF15↑	[132]
Sirt1 KO	Sirt1	UUO	Pro-fibrotic; ROS↑, COX2↓, tubular cell apoptosis↑	[119]
Sirt1 KO	Sirt1	UUO	Pro-fibrotic; HIF-2α↑	[121]
Sirt3 KO	Sirt3	Diabetic mice	Pro-fibrotic; HIF-1α↑, PKM2↑, glycolysis↑	[126]
Sirt3 KO	Sirt3	UUO	Pro-fibrotic; PDHE1α acetylation↑, EMT↑	[128]
OSS, Sirt6 KO	Sirt6	UUO	Pro-fibrotic; β-catenin↑, H3K56ac↑, ECM↑	[131]

Abbreviations: Trichostatin A (TSA); Colony Stimulating Factor 1 (CSF-1); Unilateral ureteral obstruction (UUO); diabetic nephropathy (DN); Bone Morphogenetic Protein 7 (BMP-7); extracellular matrix (ECM); aristolochic acid nephropathy (AAN); nuclear factor kappa B (NF-κB); Nuclear Receptor Corepressor 1 (Ncor); Peroxisome Proliferator-Activated Receptor Gamma (PPARγ); epithelial–mesenchymal transition (EMT); Transforming Growth Factor Beta (TGF-β); Epidermal Growth Factor Receptor (EGFR); Phosphorylated transducer and activator of transcription-3 (p-STAT3); NLR Family Pyrin Domain-Containing 3 (NLRP3); KLF Transcription Factor 15 (KLF15); Sirtuin 1 (Sirt1); Sirtuin 3 (Sirt3); Sirtuin 6 (Sirt6); reactive oxygen species (ROS); Mitochondrially Encoded Cytochrome C Oxidase II (COX2); Hypoxia-inducible factor-2α (HIF-2α); Pyruvate Kinase M1/2 (PKM2); Pyruvate Dehydrogenase E1 Subunit Alpha 1 (PDHE1α); catenin Beta 1 (β-catenin); diabetic kidney disease (DKD).
